# Auditory attention in individuals with tinnitus^☆^

**DOI:** 10.1016/j.bjorl.2019.01.011

**Published:** 2019-03-08

**Authors:** Daviany Oliveira Lima, Aline Menezes Guedes Dias de Araújo, Fátima Cristina Alves Branco-Barreiro, Cláudia da Silva Carneiro, Larissa Nadjara Alves Almeida, Marine Raquel Diniz da Rosa

**Affiliations:** a Universidade Federal da Paraíba (UFPB), Programa Associado de Pós-graduaçao em Fonoaudiologia, João Pessoa, PB, Brazil; b Universidade Federal da Paraíba (UFPB), Departamento de Fonoaudiologia, João Pessoa, PB, Brazil; c Universidade Federal de São Paulo (Unifesp), Escola Paulista de Medicina (EPM), Departamento de Fonoaudiologia, São Paulo, SP, Brazil; d Universidade Federal da Paraíba (UFPB), Modelos de Decisão em Saúde, João Pessoa, PB, Brazil; e Universidade Federal da Paraíba (UFPB), Departamento de Fonoaudiologia, Programa Associado de Pós-graduaçao em Fonoaudiologia, João Pessoa, PB, Brazil; f Grupo de Estudos e Pesquisas em Audição, Equilíbrio e Zumbido (GEPAEZ), João Pessoa, PB, Brazil

**Keywords:** Hearing, Auditory perception, Tinnitus, Auditory attention, Audição, Percepção auditiva, Zumbido, Atenção auditiva

## Abstract

**Introduction:**

Tinnitus is characterized by the presence of a sound in the absence of external sound stimulus. In individuals with normal audiometry, it may be associated with auditory attention difficulty, especially in those who report high tinnitus annoyance.

**Objective:**

To investigate auditory attention ability in individuals with tinnitus complaint.

**Methods:**

Cross-sectional analytical observational study. We evaluated 30 volunteers with normal hearing (up to 25 dBHL): 15 with tinnitus (test group) and 15 with no complaints (control group), aged between 18-40 years. The volunteers answered the tinnitus handicap inventory questionnaire and a visual analogue scale. Subsequently, a basic audiological evaluation (meatoscopy, tonal and vocal audiometry, and imittanciometry) and psychoacoustic measures of tinnitus (loudness and pitch) were performed. To evaluate auditory attention, the following tests were performed: auditory cognitive evoked potential (P300), central auditory processing tests (dichotic digits test and speech-in-noise test) and sustained auditory attention ability test.

**Results:**

In the tinnitus handicap inventory, individuals with tinnitus had a mean score of 37.78 (±27.05), characterized as moderate degree. In the dichotic digits test (binaural separation), a difference was observed between the groups in both ears. Moreover, there was a difference in the speech-in-noise test in both ears (RE: *p* = 0.044; LE: *p* = 0.019), in P300 (*p* = 0.049) and in total sustained auditory attention ability test (*p* = 0.032). Also, there is a negative correlation between sustained auditory attention ability test, decrease in attentiveness and binaural integration (RE: *p* = 0.044; LE: *p* = 0.048).

**Conclusions:**

Individuals with tinnitus had a poorer performance compared to the control group regarding auditory attention ability. Therefore, it is inferred that tinnitus is associated with poor performance in selective and sustained auditory attention in the assessed volunteers. These aspects should be considered for the management of patients with tinnitus.

## Resumo

**Introdução:**

O zumbido caracteriza-se pela presença de som na ausência de estímulo sonoro externo. Em indivíduos com audiometria normal pode estar associado à dificuldade na atenção auditiva, principalmente naqueles que referem alto incômodo do zumbido.

**Objetivo:**

Investigar a habilidade de atenção auditiva em indivíduos com queixa de zumbido.

**Método:**

Pesquisa do tipo observacional analítica de caráter transversal. Foram avaliados 30 voluntários com audição normal (até 25 dBNA): 15 com zumbido (grupo teste) e 15 sem a queixa (grupo controle), entre 18 e 40 anos. Os voluntários responderam o questionário do handicap do zumbido ‒ *Tinnitus Handicap Inventory* ‐ e a escala visual analógica. Em seguida, fez-se a avaliação audiológica básica (meatoscopia, audiometria tonal, vocal e imitanciometria) e medidas psicoacústicas do zumbido (*loudness* e *pitch*). Para avaliar a atenção auditiva, foram usados os testes: potencial evocado auditivo cognitivo (P300), processamento auditivo central (dicótico de dígitos e de fala no ruído) e o de atenção auditiva sustentada.

**Resultados:**

No *Tinnitus Handicap Inventory*, indivíduos com zumbido apresentaram escore médio de 37,78(±27,05); caracterizado por grau moderado. No teste dicótico de dígitos (separação binaural) percebeu-se diferença entre os grupos, nas duas orelhas. Além disso, houve diferença no teste de fala no ruído, em ambas as orelhas (OD: *p* = 0,044; OE: *p* = 0,019), no P300 (*p* = 0,049) e no teste de atenção auditiva sustentada total (*p* = 0,032). Além de correlação negativa entre o teste de atenção auditiva sustentada, decréscimo de vigilância e integração binaural (OD: *p* = 0,044; OE: *p* = 0,048).

**Conclusão:**

Os indivíduos com zumbido apresentaram desempenho inferior ao grupo controle na habilidade de atenção auditiva. Portanto, infere-se que o zumbido está associado ao baixo desempenho na atenção auditiva seletiva e sustentada nos voluntários pesquisados. Tais aspectos devem ser considerados para o manejo do paciente com zumbido.

## Introduction

Tinnitus is characterized by the perception of one or more sounds in the ears or head in the absence of an external acoustic signal.^1^, ^2^ It is a symptom that affects approximately 10–15% of the adult world population. In Brazil, it is believed that more than 28 million individuals have tinnitus, which makes it a public health problem.^3^ An epidemiological study carried out in the city of São Paulo shows that 22% of the population has this symptom.^4^

Due to its multifactorial etiology, tinnitus is considered a difficult symptom to treat.^1^ Otological and neurological problems, infectious diseases, medications, dental and psychological disorders can cause tinnitus.^5^

In approximately 80% of cases, tinnitus is mild and intermittent, with no major consequences for the individual's life. However, when the tinnitus manifestation is profound, it can significantly impair quality of life, affecting sleep, concentration, attention, emotional balance and even social interaction, preventing individuals from effectively performing activities of daily living.^6^

Tinnitus may be present in individuals with normal audiometry or hearing loss.^7^, ^8^ When present in individuals with normal audiometry, the high emotional implication of severe tinnitus could lead to a high level of attention directed to the symptom, which can increase the inattention and/or prevent habituation to it.^9^ Some authors report that in patients with highly annoying tinnitus, the interference with activities that demand attention is higher.^10^, ^11^

It is believed that the networks associated with attention, memory, distress, and multisensory experience are involved with the tinnitus perception.^9^ This perception can be modulated by the dorsolateral prefrontal cortex, which plays a role in attention, in the limbic system and in the secondary auditory cortex.^11^

A study has shown that selective attention in individuals with tinnitus differs from that in normal individuals.^12^ However, another study did not observe any tinnitus interference in selective attention and temporal resolution abilities using auditory processing tests (Speech-in-White Noise-Test, Dichotic Digits Test and Gaps-in-Noise Test).^13^

The literature shows a higher occurrence of alterations in the long-latency auditory evoked potentials (LLAEP) in individuals complaining of tinnitus when compared to individuals without the complaint. Additionally, patients with severe tinnitus fail to adequately habituate to the sound stimulus in the LLAEP.^9^

In clinical practice, some patients complaining of tinnitus have reported attention and concentration difficulties during activities of daily living. Based on these problems and the scarcity of studies related to tinnitus and auditory attention, it becomes important to verify whether there is an association between auditory attention difficulties and the tinnitus symptom. Moreover, the results obtained in the present study can provide measures for the control and prevention of these alterations and can be used as the basis for other studies, thus contributing to improve the quality of life of these patients.

Little is known about the electrophysiological characteristics in patients with tinnitus, and even less is known about the interactions between attention mechanisms and tinnitus. Therefore, does the tinnitus interfere with the performance of tests that evaluate auditory skills? Considering the above, the aim of this study is to investigate the auditory attention ability in individuals with tinnitus complaints and to verify whether there is a correlation with the degree of tinnitus annoyance.

## Method

This is a cross-sectional observational/descriptive study. Thirty volunteers were recruited: 15 complaining of tinnitus (test group) and 15 with no tinnitus complaint (control group), aged between 18 and 40 years. The study participants were selected at the Tinnitus Service offered at a school clinic, according to the following eligibility criteria: having unilateral or bilateral tinnitus for more than 6 months (chronic); having normal (up to 25 dBHL at all frequencies) and symmetrical hearing.^14^ The small number of volunteers in the sample was due to a shortage of individuals with tinnitus and hearing within the normality range aged between 18 and 40 years.

To meet the study objectives, the procedures were performed according to the stages described below.

### Stage I: anamnesis, audiological and tinnitus evaluation

Initially, according to Resolution 466/2012, the study was approved by the Research Ethics Committee (protocol n. 0129/12) and the volunteers who accepted to participate in the study signed the Free and Informed Consent Form. Subsequently, data collection was started through the anamnesis, basic audiological evaluation (meatoscopy, tonal/vocal audiometry and immittance audiometry), otoacoustic emissions and, after that, the application of the Tinnitus Handicap Inventory (THI), Visual Analog Scale (VAS) and psychoacoustic measures for tinnitus evaluation.

The anamnesis consisted of seven objective questions concerning hearing and tinnitus: time of tinnitus, sound description, location, type and beginning. The meatoscopy identified the presence or absence of any impediment in the external ear that could interfere with the auditory exams. In cases of impediment, the patients were referred to otorhinolaryngological evaluation and, when the impediment was corrected, they returned to undergo the other procedures.

**Figure 1:**
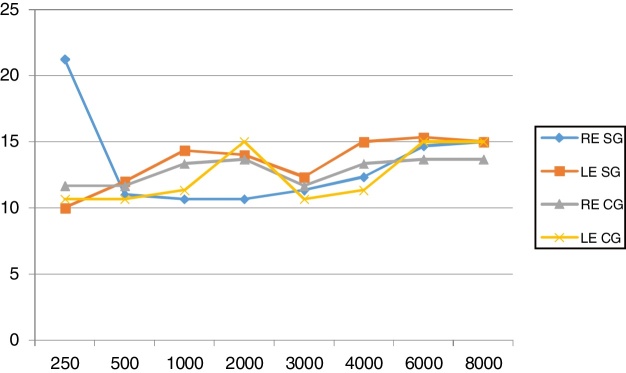
Means of hearing thresholds by frequency. RE, right ear; LE, left ear; SG, study group; CG, control group.

The audiological evaluation was performed using an Interacoustics® AD229 two-channel audiometer in an acoustic booth. Individuals with normal hearing were those who had an auditory threshold ≤25 dBHL (decibel Hearing Level).^14^ The means are shown in Fig. 1. To perform the acoustic immittance measurements, an Interacoustics® AT 235 middle-ear analyzer was used to evaluate middle-ear compliance and the acoustic stapedial reflex. Only individuals with type “A” tympanogram and acoustic reflexes were included in the sample.

Finally, the following protocols were applied: the THI, consisting of a self-assessment questionnaire aimed to quantify the impact of tinnitus on quality of life.^15^, ^16^ The THI consists of 25 questions, of which answers can be “yes” (4 points), “no” (0 point) or “sometimes” (2 points), and each question is related to one of the domains: functional, emotional or catastrophic. After that, the EVA was applied, consisting of a graphic-visual form to determine the perceived annoyance generated by tinnitus, quantifying it on a scale of 0–10.^14^

When performing the psychoacoustic measures, according to the type of tinnitus reported by the patient during the anamnesis, the pure tone (continuous, pulsatile and Frequency Modulated), narrow band noise and the white noise were presented to the ear contralateral to the tinnitus. In cases of bilateral tinnitus, the stimulus was presented to the ear contralateral to the most intense tinnitus.

Subsequently, the tinnitus frequency sensation (pitch) was investigated in the contralateral ear. The frequencies of 8000 Hz and 500 Hz were compared, and, after the choice, other frequencies were then investigated until the patient identified the tinnitus frequency. To investigate the tinnitus intensity sensation (Loudness), at the previously estimated tinnitus frequency and at its auditory threshold, the study was performed in the ipsilateral ear of the tinnitus reported by the patient, testing every 5 dBHL, and then through the 1 dBHL scale at the frequency pre-determined by the patient. It was considered the value in dBSL (dB sensation level), that is, the value obtained subtracted from the patient's auditory threshold at the tinnitus frequency sensation (pitch).^17^

### Stage II – behavioral and electrophysiological evaluation

Individuals who met the study eligibility criteria were referred to the second stage of the study. At this stage, the electrophysiological (long-latency auditory evoked potentials – P300) and auditory processing behavioral tests were performed (Dichotic Digits Test – Integration and Separation, Speech-in-Noise Test, and the Sustained Auditory Attention Ability Test – SAAAT). Each of them will be discussed in details below.

To evaluate the ability of sustained auditory attention, the P300 (Short-Latency Auditory Evoked Potential) was used, through the dual-channel SmartEP equipment, after cleaning the skin with 70% alcohol and after the electrodes were fixed to the subject's skin using the electrode electrolyte paste, in the positions M1 (left mastoid) and M2 (right mastoid), Cz (vertex), with the ground electrode (Fpz) being placed on the forehead. The acoustic stimulus was presented through insert earphones, eliciting the responses. The patients were instructed to pay attention to the different stimuli (rare stimulus) that appeared randomly, within a series of equal stimuli (frequent stimulus) (Table 1).

**Table 1:** Parameters used to obtain P3 potential.^18^

Intelligent Hearing System® equipment	SmartEP module
Electrodes: M1, M2, Fpz and Cz	Electrode impedance ≤3 kohms
Intensity 75 dB Pe	Stimulation type: binaural
Number of stimuli: 300 (80% frequent and 20% rare)	Channels: AB
Velocity: 0.8 pps	Duration: 2.0 ms
Phase	Alternate
Stimulus utilized	1 kHz (frequent) 4 kHz (rare)
Transducer type	Insert earphones
Stimulus duration	50,000 μs
Envelope	Trapezoid
Individual status	Alert

kohms, kiloohms; dBHL, decibel in hearing level; pps, pulses per second; ms, milliseconds; kHz, kilohertz; μs, microseconds.

Subsequently, the behavioral tests were performed to evaluate the selective and sustained attention of the individuals participating in the study. For this purpose, the following tests were used: Dichotic Digits (DD) Test (binaural integration/separation) and monotonic speech-in-noise test.

The DD test used a list consisting of 20 pairs of digits representing Portuguese language disyllables (4, 5, 7, 8 and 9). To evaluate the binaural integration ability, two pairs of digits were presented at each ear simultaneously, and the patients were asked to repeat orally all the digits they heard, regardless of the order.^19^

Then, the speech-in-noise test was performed using a list consisting of 25 mono-syllables and a competitive message consisting of white noise, at a signal-to-noise ratio of (+5 dB). When applying the test, the monosyllables and white noise were presented simultaneously and ipsilaterally, and the patients were asked to orally repeat the monosyllables they heard.^19^

All of the abovementioned tests were performed at an intensity of 50 dBSL. The measurements were obtained in an acoustic booth, using properly calibrated auditory processing equipment (Acústica Orlandi®, model PA2004), and type TDH39 earphones (Acustica Orlandi®). The stimuli were presented using an Ipod device (Apple^TM^) coupled to the auditory processing equipment.

In order to evaluate the sustained auditory attention, we used the Sustained Auditory Attention Ability Test – SAAAT, which is based on the ACPT – Auditory Continuous Performance Test, clinically used to measure auditory attention.^16^ The test was presented in a dichotic form, that is, the same information was presented concomitantly to both ears, through earphones. The test consists of the presentation of a list of 21 monosyllabic words, accessed through the website and coupled to the Acustica Orlandi® equipment; these words were presented six times without interruption, totaling 600 words throughout the test.^16^ Each participant received verbal instructions given by the evaluator: they would hear a list of words and should raise their hand every time they heard the word “NO”. The test was applied by the researcher herself and lasted approximately 10 min.

As for the SAAAT performance, it considers the total score of errors and the decrease of attentiveness. The total error score is obtained by adding the Inattention number (In) plus the number of Impulsivity (I). In the SAAAT, Inattention is an error when the individuals do not raise their hand when they hear the word “NO” before the next word is presented, and Impulsivity is when the individuals raise their hand to another word instead of the word “NO”. The decreased attentiveness, that is, the decline in attention that occurs over time during the attention task, is obtained by calculating the number of correct answers for the word “NO” at the first presentation and the number of correct responses for the 6th presentation. The difference between these two numbers is what is called decreased attentiveness.^20^

The data were categorized and added to a digital spreadsheet. The variables were descriptively – mean, standard deviation and frequency measures – and inferentially analyzed – tests: *t*-Student for independent samples and Spearman's correlation. The statistical software R, version 2.11.0, was used, with significance level of 5%.

## Results

The Study Group (SG) participants had a mean age of 25.40 (±7.36) years, and most were males (53.3%, *n* = 7) and students (53.3%, *n* = 7). The control group (CG) had a mean age of 26.66 ± 7.06 years, most were females (60.0%, *n* = 9) and students (73.3%, *n* = 11). The sample was homogeneous.

Table 2 shows data characterizing the volunteers’ tinnitus. There was a predominance of tinnitus in the left ear (33.3%; *n* = 5), single type (60.0%; *n* = 9), with sudden onset (60.0%; *n* = 9), constant (73.3%, *n* = 73.3) and with a whistling sound (40.0%, *n* = 6).

**Table 2:** Tinnitus characterization in study group volunteers.

Variable	Study group	
	*n*	%
*Location*		
RE	4	26.7
LE	5	33.3
R = L	3	20.0
R > L	3	20.0
R < L	0	0.0
Head	0	0.0

*Type*		
Single	9	60.0
Multiple	3	20.0
Pulsatile	2	13.3
Single click	1	6.7

*Start*		
Progressive	6	40.0
Sudden	9	60.0

*Perception*		
Constant	11	73.3
Intermittent	4	26.7

*Description of the sound*		
Whistling	9	60.0
Wheezing	4	26.7
Pulsatile	2	13.3

*Acuphenometry stimulus RE*		
Continuous	9	60.0
Modulated frequency	0	0.0
NB	2	13.3
Did not report tinnitus in the RE	4	26.7

*Acuphenometry stimulus LE*		
Continuous	10	66.7
Modulated frequency	1	6.7
NB	0	0.0
Did not report tinnitus in the LE	4	26.7

It was observed that the individuals reported the symptom occurrence for 5.04 (±6.20) years, on average, with a value of 6.21 (±1.84) on the visual-analog scale. They had a mean score of 37.78 (±27.05) in the total THI, characterizing a moderate degree, especially in the Functional domain, with a mean score of 17.85 (±12.97) points (Table 3).

**Table 3:** Mean and standard deviation of tinnitus variables in the test group individuals.

Variables	Study group	
	Mean	SD
Time of tinnitus	5.04	6.20
VAS	6.21	1.84
THI: Functional	17.85	12.97
THI: Emotional	13.35	10.62
THI: Catastrophic	9.14	6.16
Total THI	37.78	27.05
Psychoacoustic measure: frequency RE	3000	2576.45
Psychoacoustic measurement: intensity RE	13	11.96
Psychoacoustic measure: frequency LE	3000	3057.29
Psychoacoustic measure: intensity LE	13	15.00

SD, standard deviation; VAS, Visual Analog Scale; RE, right ear; LE, left ear; THI, Tinnitus Handicap Inventory.

In the psychoacoustic measures, it was observed that the tinnitus mean frequency sensation in the right ear was 3000 Hz (±2576.45) and in the left ear, 3000 Hz (±3057.29), with a mean intensity sensation of 13 (±11.96) and 13 (15.00), respectively.

Table 4 shows the behavioral and electrophysiological evaluation of the study and control groups. Differences were observed in the binaural separation of the Dichotic Digits test, both in the right (*p* = 0.009) and in the left (*p* = 0.001) ears, being statistically significant. There was also a difference between the results of the Speech-in-Noise test in both ears (RE: *p* = 0.044, LE: *p* = 0.019), P300 (*p* = 0.049) and Total SAAAT (*p* = 0.032).

**Table 4:** Mean, standard deviation and comparison of behavioral and electrophysiological evaluation between the study and control groups.

Variable	Study group		Control group		*p*-Value
	Mean	SD	Mean	SD	
*Dichotic Digits Test*					
Binaural integration RE	95.89	6.91	97.66	2.90	0.087
Binaural integration LE	98.00	4.83	95.40	5.07	0.508
Binaural separation RE	96.50	8.18	100.00	0.000	0.009^a^
Binaural separation LE	94.60	7.29	97.66	3.33	0.001^a^

*Speech-in-Noise Test*					
PSRI RE	95.60	5.25	96.13	3.41	0.262
PSRI LE	96.26	3.53	94.00	3.46	0.563
SN RE	82.93	13.13	86.93	9.25	0.044^a^
SN LE	81.33	11.17	85.60	7.97	0.019^a^
P300	305.86	37.68	301.40	23.28	0.049^a^
Total SAAAT	3.26	5.86	1.00	1.30	0.032^a^
SAAAT decrease	0.86	1.18	0.46	0.74	0.152

SD, standard deviation; PSRI, Percentage of Speech Recognition Index; SN, Speech-in-Noise; RE, right ear; LE, left ear; SAAAT, Sustained Auditory Attention Ability Test.

a Student's *t*-test – independent samples; *p* < 0.05.

A positive correlation was observed between total THI scores with VAS (*p* = 0.036) and SAAAT responses (*p* = 0.041). There was also a negative correlation between SAAAT, decrease in attentiveness and binaural integration in the right (*p* = 0.044) and left (*p* = 0.048) ears (Table 5).

**Table 5:** Correlation between self-assessment results and auditory aspects of individuals in the study group.

Variable	Test statistics	*p*-Value^a^
Total THI × VAS	0.563	0.036
Total THI × SAAAT	0.482	0.041
SAAAT AD × binaural integration RE	−0.546	0.044
SAAAT AD × binaural integration LE	−0.454	0.048

VAS, Visual Analog Scale; SAAAT, Sustained Auditory Attention Ability Test; AD, attentiveness decrease; THI, Tinnitus Handicap Inventory; RE, right ear; LE, left ear.

a Pearson's Correlation Test; *p* < 0.05.

## Discussion

The human ear has a restricted ability to process the arrival of a given stimulus.^21^ Therefore, it is considered that the attentional mechanisms are important to limit the amount of processed information.

Concerning tinnitus and attentional issues, more specifically in relation to auditory attention, studies have shown that individuals have concentration and attention difficulties due to tinnitus.^7^, ^22^ In the present study, subjects with tinnitus reported a moderate degree of annoyance, a fact that can hinder concentration. Another study that used the THI questionnaire also showed that most patients had concentration and sustained attention difficulties.^23^

Regarding the binaural separation of the dichotic digits test, a better result was observed in both ears in the two tests for the non-tinnitus group. There was also a difference between the results of the Speech-in-Noise test in both ears. These findings suggest that tinnitus may be disrupting these individuals’ selective attention. Studies have directly assessed the impact of tinnitus on selective attention and have concluded that it has an effect on the cognitive performance by disrupting selective attention.^9^, ^22^, ^23^

The present study showed a positive correlation between the total THI scores with the VAS and SAAAT responses, suggesting that the greater the impact of tinnitus on the individual's life, the greater the annoyance and interference of tinnitus with sustained attention (concentration).

To date, no study has been found in the literature that evaluated the sustained attention ability using SAAAT in tinnitus patients, but considering the studied population, it can be inferred that individuals with the symptom have greater difficulties in concentration. There was a negative correlation between SAAAT, decrease in attentiveness and binaural integration, and right and left ears; that is, the greater the number of errors in the SAAAT (the worse the sustained attention), the lower the binaural integration value (more errors).

Regarding the mean P300 latency, a statistically significant difference was observed between both groups. Such findings corroborate the evaluated literature,^24^ in which latency differences were observed in individuals with and without tinnitus; that is, in individuals with tinnitus, the latency is increased.

Individuals with tinnitus commonly complain of difficulties in concentration and in activities of daily living.^25^ The components of LLAEP are influenced by the degree of attention to the stimulus. If the stimulus is ignored, the waveforms are attenuated and possibly delayed.^26^

It is also believed that tinnitus has a masking effect on the acoustic signals presented to these individuals.^27^ Therefore, it can be inferred that individuals in the test group were less attentive during the tests, probably due to the presence of tinnitus and, consequently, the reduced attention could have been a contributing factor to the increase of P300 latency.^25^

The alterations in P300 observed in individuals with tinnitus demonstrate the involvement of the Central Auditory System, suggesting the participation of the auditory cortex in the tinnitus generation and/or maintenance.^28^

A study has shown that the reaction time for the rare stimulus presented, observed in patients with tinnitus, was significantly slower than in the control group (no tinnitus).^23^ Another study^8^ evaluated the selective attention in individuals with tinnitus using the Stroop Test, and concluded that the reaction time in the tinnitus group was slower (1559 ms) than in the other group.

Other possible factors that can be attributed to the increased P300 wave latency in individuals complaining of tinnitus are the possibility of a reduction in the number of functioning neurons, a decrease in neural activity and/or greater firing desynchronization in the affected neurons.^29^

In summary, based on the data from this study, it can be inferred that tinnitus is a factor that can disperse the selective and sustained attention of these individuals. This fact can interfere with the activities of daily living and be an impediment factor for the habituation mechanism. Additionally, the behavioral and electrophysiological tests are important in identifying cognitive (attention) aspects in individuals complaining of tinnitus.

## Conclusion

Tinnitus with a moderate degree of annoyance was observed. The binaural integration ability performance in subjects with tinnitus was worse than that of the control group. In addition, selective and sustained auditory attention was found to be diminished in these subjects when compared to control group.

Therefore, it is important to consider the attentional processes during the evaluation of these individuals, since the behavioral and electrophysiological tests are important in the identification of cognitive aspects (attention) in these patients and such factors may be important to facilitate the management of tinnitus.

## Conflicts of interest

The authors declare no conflicts of interest.
